# Comparative Evaluation of Smear Layer Removal Efficacy of Ethylenediaminetetraacetic Acid (EDTA) Gel Versus Solution With Sonic Activation: A Scanning Electron Microscopic Study

**DOI:** 10.7759/cureus.109951

**Published:** 2026-05-30

**Authors:** Gurkirat Singh, Rajinder Bansal, Manu Bansal, Jasvinder Kaur, Sangam Mittal, Nishant Sharma

**Affiliations:** 1 Department of Conservative Dentistry and Endodontics, Gian Sagar Dental College, Banur, IND; 2 Department of Conservative Dentistry and Endodontics, Guru Nanak Dev Dental College and Research Institute, Sunam, IND

**Keywords:** endodontics, root canal irrigation, scanning electron microscopy, smear layer, sonic activation

## Abstract

Introduction: During root canal instrumentation, a smear layer forms on the dentinal walls, which may hinder effective disinfection. Ethylenediaminetetraacetic acid (EDTA) is commonly used to remove this layer through calcium ion chelation. This study aims to compare the smear layer removal efficacy of EDTA gel and EDTA solution when used with sonic activation, evaluating their performance in the coronal, middle, and apical thirds of single-rooted human teeth using scanning electron microscopy (SEM).

Methodology: Sixty freshly extracted single-rooted human teeth were randomly allocated into four groups (n = 15 each): Group 1 (17% EDTA gel), Group 2 (19% EDTA gel), Group 3 (17% EDTA solution), and Group 4 (18% EDTA solution). Following standardized biomechanical preparation to size #30/.04 taper using NeoEndo rotary files (Orikam Healthcare India Pvt. Ltd., Haryana, India) and 5% sodium hypochlorite (NaOCl) irrigation, each assigned irrigant (5 mL) was delivered with a 30-gauge side-vented needle and immediately activated using a sonic activator system (25/04 tip, 10,000 rpm) for one minute. Specimens were longitudinally sectioned, sputter-coated, and examined by SEM at ×1000 magnification. Smear layer presence at coronal, middle, and apical thirds was scored. Data were analyzed by one-way ANOVA and post-hoc Tukey’s test (p < 0.05).

Results: Statistically significant intergroup differences in mean smear layer scores were observed (F = 24.10, p = 0.001). Group 4 (18% EDTA solution) demonstrated the lowest mean score (1.48 ± 0.30), indicating superior smear layer removal, while Group 1 (17% EDTA gel) recorded the highest mean score (2.55 ± 0.34), reflecting the poorest efficacy. Post-hoc Tukey analysis confirmed significant differences between solution and gel groups (p < 0.001). Segment-wise analysis revealed the greatest intergroup variation at the apical third (F = 31.48, p < 0.001). Intragroup analysis demonstrated statistically significant deterioration of cleaning efficacy from coronal to apical in gel groups (Group 1: p = 0.004; Group 2: p < 0.001), whereas solution groups showed uniform performance across all thirds (p > 0.05).

Conclusion: Within the limitations of this in vitro study, EDTA solution formulations were significantly more effective than gel formulations in smear layer removal under sonically activated conditions. The 18% EDTA solution provided the most consistent and superior cleaning across all canal thirds, particularly at the apical level, suggesting that solution formulations should be preferred when sonic activation protocols are employed.

## Introduction

Effective smear layer removal is a fundamental prerequisite for successful endodontic therapy. The smear layer, a microcrystalline, amorphous film deposited on instrumented root canal walls, is generated as an inevitable by-product of chemomechanical preparation and consists of organic debris, inorganic dentinal particles, remnants of odontoblastic processes, pulpal tissue, and microbial elements [1]. Its presence occludes dentinal tubules, impairs irrigant and medication penetration into the dentinal microstructure, and compromises the adaptation of obturating materials to canal walls, all of which collectively threaten the long-term outcome of root canal treatment. The clinical significance of complete smear layer elimination has therefore been firmly established in contemporary endodontic literature [2,3].

Ethylenediaminetetraacetic acid (EDTA) has remained the most widely advocated chelating agent for smear layer dissolution since its initial introduction into clinical endodontics by Nygaard-Östby in 1957 [4]. EDTA exerts its action through chelation of calcium ions within the inorganic dentinal matrix, thereby disrupting hydroxyapatite crystalline architecture and rendering the smear layer amenable to removal. It is routinely employed as a final rinse in conjunction with sodium hypochlorite (NaOCl), which addresses the organic component of the smear layer, to achieve comprehensive debridement of both organic and inorganic constituents [5]. EDTA is commercially available in two principal formulations, aqueous solution and viscous gel, each distinguished by its rheological properties, surface tension characteristics, and, consequently, its interaction with canal wall surfaces. While the solution form disperses readily throughout the canal system, the gel formulation exhibits greater substantivity, maintaining contact with dentinal surfaces over extended periods, which theoretically enhances chelating efficacy [6].

Considerable variation exists in the literature regarding the optimal concentration of EDTA for clinical use. The most extensively studied and internationally accepted concentration is 17%, which has demonstrated consistent smear layer removal across coronal, middle, and apical thirds of the root canal system [7]. However, investigators have explored both lower and higher concentrations in an effort to improve chelating efficiency and reduce the contact time required for adequate smear layer elimination [8]. Concentrations ranging from 15% to 19% have been examined with variable outcomes, and the comparative efficacy of gel versus solution formulations at equivalent or differing concentrations remains insufficiently characterized. Furthermore, the influence of sonic activation on irrigant efficacy has received growing attention, as passive delivery of irrigants is widely recognized to result in suboptimal fluid dynamics, particularly in the apical third, where anatomical complexity limits irrigant exchange [7].

Sonic activation, achieved through devices such as the EndoActivator (SmartLite Pro EndoActivator, Dentsply Sirona, North Carolina, USA) or equivalent systems, imparts acoustic streaming and cavitation forces to the irrigant, enhancing its penetration into lateral canals, isthmuses, and dentinal tubules [9,10]. When applied to chelating agents, sonic activation is postulated to potentiate smear layer removal by facilitating more uniform distribution and improving irrigant-wall contact. Despite this theoretical advantage, head-to-head comparative data on gel versus solution formulations of EDTA at varying concentrations under sonically activated conditions remain limited [11]. Scanning electron microscopy (SEM) provides the gold-standard morphological assessment of smear layer presence, enabling objective evaluation across canal thirds at high magnification [12]. The present study was therefore designed to compare the smear layer removal efficacy of 17% EDTA gel, 19% EDTA gel, 17% EDTA solution, and 18% EDTA solution, each delivered with one-minute sonic activation, across coronal, middle, and apical segments of single-rooted human teeth, as evaluated by SEM at ×1000 magnification and scored using Torabinejad's validated criteria [13].

## Materials and methods

Study design and ethical considerations

This in vitro study was conducted from November 2018 to December 2019 in the Department of Conservative and Endodontics at Guru Nanak Dev Dental College and Research Institute, Sunam, following clearance by the Institutional Ethics Committee (GNDDC/2018/306). All procedures were carried out in accordance with the principles of the Declaration of Helsinki. Informed consent was obtained from patients from whom extracted teeth were collected, and teeth were handled according to standard biosafety protocols.

Sample size estimation

Sample size calculation was performed using G*Power software (version 3.1, Heinrich Heine University, Düsseldorf, Germany). Adopting an F test family, an omnibus one-way comparison with an effect size (Cohen's f) of 0.45 derived from a previous study [8] analyzing different EDTA concentrations on root surface smear removal, a significance level (α) of 0.05, and statistical power of 0.80, the minimum required sample size was calculated as 15 subjects per group. Therefore, a total of 60 teeth (15 teeth per group) across four groups were examined.

Sample selection

A total of 60 freshly extracted single-rooted human teeth were collected for this in vitro experimental study. Inclusion criteria comprised teeth with fully formed apices, a single straight root canal with a curvature of less than 5° as determined, and the absence of calcifications, internal or external resorption, or prior endodontic treatment as verified by periapical radiography in both buccolingual and mesiodistal projections. Teeth with root cracks, fractures, or anatomical anomalies were excluded. Following extraction, teeth were stored in 0.9% normal saline at room temperature and used within four weeks.

Biomechanical preparation

Soft tissue remnants and calculus deposits were removed from all tooth surfaces using hand scalers. Each tooth was decoronated at the cementoenamel junction using a water-cooled diamond disc to standardize root length to 14 mm. Working length was established 1 mm short of the apical foramen, as confirmed radiographically. All canals were biomechanically prepared using a standardized rotary system (NeoEndo Rotary File, Orikam Healthcare India Pvt. Ltd., Haryana, India) to a final apical size of #30/.04 taper. Irrigation with 2 mL of 5% NaOCl (Prime Dental Products Pvt. Ltd., Thane, India) was performed between each instrument exchange using a 30-gauge side-vented needle to maintain canal patency and remove debris. Following the completion of instrumentation, a final irrigation sequence with 5 mL of normal saline was delivered to neutralize residual NaOCl prior to the application of experimental irrigants.

Experimental groups

The 60 specimens were randomly allocated, using a computer-generated random number sequence, into four experimental groups of 15 teeth each, according to the final irrigant applied: Group 1 (n = 15): 17% EDTA Gel (EDTA Gel, Prevest DenPro Limited, Jammu, India); Group 2 (n = 15): 19% EDTA Gel (Meta Chel Cream, META BIOMED Co., Ltd., Cheongju-si, South Korea); Group 3 (n = 15): 17% EDTA Solution (Meta Cleanser, META BIOMED Co., Ltd., Cheongju-si, South Korea); Group 4 (n = 15): 18% EDTA Solution (Ultradent EDTA Solution, Ultradent Products Inc., South Jordan, USA).

In each group, the assigned irrigant was delivered into the root canal using a 30-gauge side-vented needle positioned 2 mm short of the working length. A volume of 1 mL was deposited uniformly across all specimens. Immediately following irrigant delivery, sonic activation was performed using a sonic activator system (25/04 tip) at 10,000 cycles per minute for one minute per canal, with tip positioning at 2 mm short of working length. Following sonic activation, canals were flushed with 5 mL of distilled water to remove residual irrigant and smear layer debris.

Specimen processing and scanning electron microscopic analysis

**Figure 1:**
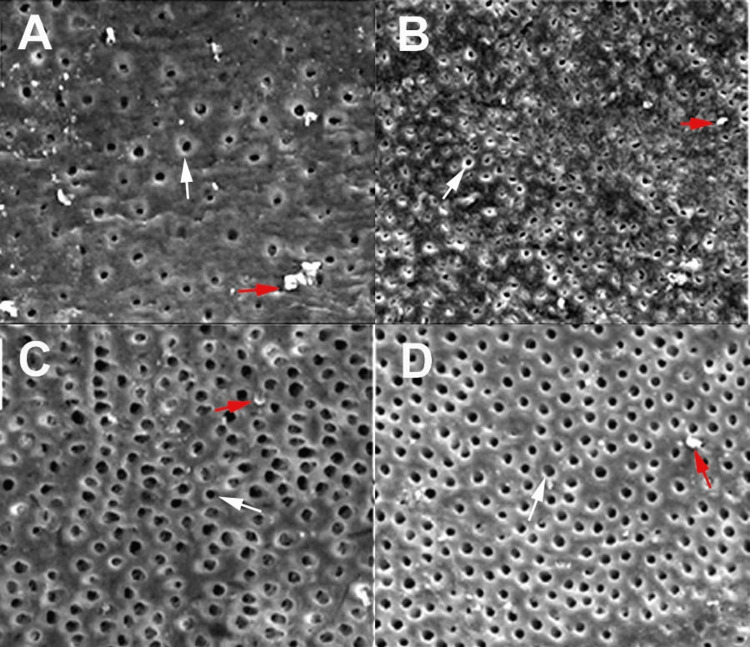
SEM images of dentin surfaces at ×1000 magnification (15 kV, working distance 9 mm; scale bar = 10 µm) showing smear layer removal following application of different EDTA formulations with sonic activation A: 17% EDTA gel (Prevest DenPro, Jammu, India) showing partial smear layer removal with few open dentinal tubules; B: 19% EDTA gel (META BIOMED Co., Ltd., Cheongju-si, South Korea) demonstrating moderate smear layer removal with increased visibility of dentinal tubules; C: 17% EDTA solution (META BIOMED Co., Ltd., Cheongju-si, South Korea) showing improved smear layer removal with more patent dentinal tubules; and D: 18% EDTA solution (Ultradent Products Inc., South Jordan, USA) exhibiting maximum smear layer removal with numerous clearly open dentinal tubules. White arrows indicate dentinal tubules, and red arrows indicate smear debris. EDTA: ethylenediaminetetraacetic acid; SEM: scanning electron microscopy

All roots were longitudinally grooved on the buccal and lingual surfaces using a water-cooled diamond disc and subsequently split into two halves using a chisel and mallet. The canal-bearing halves were mounted on aluminum stubs, sputter-coated with a gold-palladium alloy under vacuum conditions, and examined using SEM at ×1,000 magnification. Three standardized regions of each specimen were evaluated: the coronal third, measured at 9 mm from the apex; the middle third, measured at 6 mm from the apex; and the apical third, measured at 3 mm from the apex, a reference point. All SEM imaging was performed by a single calibrated examiner blinded to group allocation (Figure 1).

Smear layer scoring criteria

Smear layer presence at each evaluated region (coronal, middle, and apical) was assessed and scored using the validated four-point criteria described by Torabinejad et al. (Table 1) [13].

**Table 1:** Scoring criteria to analyze the presence or absence of the smear layer on the root canal surface or dentinal tubules The scoring criteria used in the present study were adapted from the methodology described by Torabinejad et al. [13], and the necessary permissions for use and reproduction were obtained.

Score	Category	Description
Score 1	No smear layer	No smear layer on the surface of the root canals; all tubules were clean and open.
Score 2	Moderate smear layer	No smear layer on the surface of the root canal, but the tubules contained debris.
Score 3	Heavy smear layer	The smear layer covered the root canal surface and the tubules.

Statistical analysis

All data were entered into a statistical software package, IBM SPSS Statistics for Windows, Version 26 (Released 2018; IBM Corp., Armonk, New York, United States), and analyzed at a significance level of p < 0.05. The distribution of smear layer scores across the four groups was compared using a one-way analysis of variance (ANOVA) following confirmation of normality by the Shapiro-Wilk test and homogeneity of variance by Levene's test. Where the overall ANOVA reached statistical significance, post-hoc pairwise comparisons were conducted using Tukey's honestly significant difference (HSD) test to identify inter-group differences at the coronal, middle, and apical thirds independently.

## Results

The intergroup comparison using one-way ANOVA demonstrated a statistically significant difference in mean smear layer scores among the four groups (p = 0.001) (Table 2). Group 1 (17% EDTA gel) exhibited the highest mean score, indicating the poorest smear layer removal, while Group 4 (18% EDTA solution) showed the lowest mean, reflecting superior cleaning efficacy. Post-hoc analysis using Tukey’s test (Table 3) revealed that most pairwise comparisons were statistically significant. Specifically, Group 1 showed significantly higher scores compared to Groups 3 and 4 (p < 0.001), and Group 2 also differed significantly from Groups 3 and 4. After Bonferroni correction (p < 0.008), all comparisons except Group 1 vs Group 2 remained significant, confirming that EDTA solutions performed better than gels.

**Table 2:** Intergroup comparison with one-way ANOVA test Values are expressed as mean ± standard deviation (SD). Statistical analysis was performed using one-way ANOVA to compare differences among the four groups. *p < 0.05 was considered statistically significant. N: number of samples per group; EDTA: ethylenediaminetetraacetic acid

Group	N	Mean ± SD	Min	Max	F	p-value
Group 1 - 17% EDTA gel	15	2.55 ± 0.34	2.00	3.00	24.10	0.001*
Group 2 - 19% EDTA gel	15	2.28 ± 0.45	1.33	2.67		
Group 3 - 17% EDTA solution	15	1.91 ± 0.36	1.33	2.33		
Group 4 - 18% EDTA solution	15	1.48 ± 0.30	1.00	2.00		

**Table 3:** Post-hoc analysis with Tukey test Values represent mean differences between groups. Bonferroni correction was applied, and p < 0.008 was considered statistically significant. *Indicates statistically significant difference. Group 1: 17% EDTA gel; Group 2: 19% EDTA gel; Group 3: 17% EDTA solution; Group 4: 18% EDTA solution EDTA: ethylenediaminetetraacetic acid

Comparison	Mean Difference	t-value	p-value
Group 1 vs Group 2	0.27	2.01	0.049
Group 1 vs Group 3	0.64	4.78	<0.001*
Group 1 vs Group 4	1.07	7.99	<0.001*
Group 2 vs Group 3	0.37	2.76	0.008
Group 2 vs Group 4	0.80	5.97	<0.001*
Group 3 vs Group 4	0.43	3.21	0.002*

Segment-wise comparison across the four groups revealed statistically significant differences in smear layer scores at coronal, middle, and apical levels, as demonstrated by one-way ANOVA (Table 4). The highest variation was observed in the apical segment (p < 0.001), followed by the coronal and middle segments. Across all segments, Group 1 consistently exhibited higher mean scores, whereas Group 4 showed the lowest values. Post-hoc Tukey analysis (Table 5) indicated significant differences, particularly between Groups 1 and 4, across all segments (p < 0.001). In the apical region, Groups 1 and 2 did not differ significantly, whereas both differed significantly from Groups 3 and 4. After Bonferroni correction (p < 0.008), the most consistent significant differences were observed in comparisons involving Group 4, emphasizing its superior smear layer removal.

**Table 4:** Segment-wise group comparison with ANOVA test Values are expressed as mean ± standard deviation (SD). Intergroup comparisons within each canal segment were performed using one-way ANOVA. *p < 0.05 was considered statistically significant. EDTA: ethylenediaminetetraacetic acid

Root segment	Group 1- 17% EDTA gel	Group 2- 19% EDTA gel	Group 3- 17% EDTA solution	Group 4- 18% EDTA solution	F	p-value (ANOVA)
Coronal (Mean ± SD)	2.26 ± 0.45	1.73 ± 0.45	1.66 ± 0.48	1.33 ± 0.48	13.62	0.000*
Middle (Mean ± SD)	2.53 ± 0.51	2.26 ± 0.88	2.00 ± 0.75	1.53 ± 0.51	5.74	0.002*
Apical (Mean ± SD)	2.86 ± 0.35	2.86 ± 0.35	2.06 ± 0.45	1.60 ± 0.50	31.48	0.000*

**Table 5:** Post-hoc analysis with Tukey test Values represent t-values and corresponding p-values for pairwise group comparisons within each canal segment. Bonferroni correction was applied, and p < 0.008 was considered statistically significant. *Indicates statistically significant difference. Group 1: 17% EDTA gel; Group 2: 19% EDTA gel; Group 3: 17% EDTA solution; Group 4: 18% EDTA solution EDTA: ethylenediaminetetraacetic acid

Comparison	Coronal		Middle		Apical	
	t-value	p-value	t-value	p-value	t-value	p-value
Group 1 vs Group 2	3.22	0.002*	1.09	0.28	0	1
Group 1 vs Group 3	3.48	0.001*	2.07	0.043	5.38	<0.001*
Group 1 vs Group 4	5.63	<0.001*	4.44	<0.001*	8.14	<0.001*
Group 2 vs Group 3	0.41	0.68	0.98	0.33	5.38	<0.001*
Group 2 vs Group 4	2.43	0.018	2.77	0.008	8.14	<0.001*
Group 3 vs Group 4	1.78	0.08	1.96	0.055	2.63	0.011

Intragroup analysis

Intragroup comparison using repeated measures ANOVA demonstrated significant variation in smear layer scores across canal segments in Groups 1 and 2 (Table 6). Group 2 exhibited the highest level of significance (F = 12.84, p < 0.001), followed by Group 1 (F = 6.21, p = 0.004). In contrast, Groups 3 and 4 did not show statistically significant differences across segments (p > 0.05), indicating more uniform smear layer removal. Post-hoc Bonferroni analysis (Table 7) revealed that in Groups 1 and 2, the apical segment had significantly higher scores compared to the coronal and middle segments (p < 0.001). However, no significant pairwise differences were observed within Groups 3 and 4. These findings suggest that gel formulations exhibit greater variability across segments, while solution forms provide more consistent cleaning.

**Table 6:** Intragroup comparison of smear layer removal scores across coronal, middle, and apical segments using repeated measures ANOVA Values are expressed as mean ± standard deviation (SD). *p < 0.05 was considered statistically significant. EDTA: ethylenediaminetetraacetic acid

Groups	Coronal (Mean ± SD)	Middle (Mean ± SD)	Apical (Mean ± SD)	F	p-value
Group 1- 17% EDTA gel	2.26 ± 0.45	2.53 ± 0.51	2.86 ± 0.35	6.21	0.004 *
Group 2- 19% EDTA gel	1.73 ± 0.45	2.26 ± 0.88	2.86 ± 0.35	12.84	< 0.001*
Group 3- 17% EDTA solution	1.66 ± 0.48	2.00 ± 0.75	2.06 ± 0.45	2.31	0.11
Group 4- 18% EDTA solution	1.33 ± 0.48	1.53 ± 0.51	1.60 ± 0.50	1.84	0.17

**Table 7:** Post-hoc analysis with Bonferroni test Values represent t-values and corresponding p-values for pairwise comparisons between coronal, middle, and apical segments within each group. *p < 0.05 was considered statistically significant. EDTA: ethylenediaminetetraacetic acid

Comparison	Group 1- 17% EDTA gel		Group 2- 19% EDTA gel		Group 3- 17% EDTA solution		Group 4- 18% EDTA solution	
	t-value	p-value	t-value	p-value	t-value	p-value	t-value	p-value
Coronal vs Middle	1.58	0.12	2.05	0.045*	1.53	0.13	1.21	0.23
Coronal vs Apical	3.64	<0.001*	6.47	<0.001*	2.39	0.06	1.77	0.08
Middle vs Apical	2.06	0.045*	3.72	<0.001*	0.29	0.77	0.39	0.69

## Discussion

The present study evaluated the smear layer removal efficacy of two EDTA formulations (gel and solution) at varying concentrations, each delivered with one-minute sonic activation, across coronal, middle, and apical thirds of single-rooted human teeth. The principal finding was that an 18% EDTA solution (Group 4) demonstrated statistically superior smear layer removal compared to all other groups, while a 17% EDTA gel (Group 1) exhibited the poorest performance. Overall, solution formulations outperformed gel formulations across all canal thirds (p < 0.001), and apical third cleaning remained the greatest challenge regardless of the irrigant used.

The superior performance of EDTA solution over gel formulations in this study is consistent with the rheological properties of each vehicle. Aqueous solutions exhibit lower viscosity and surface tension, enabling more uniform distribution through the canal lumen and greater penetration into dentinal tubules, particularly in the constricted apical third, where irrigant exchange is anatomically limited. Putzer et al. demonstrated that EDTA efficacy depends critically on concentration, pH, contact time, and the physical state of the irrigant, with solution forms showing superior diffusion kinetics compared to viscous gels [14]. The recommendation by Jaju and Jaju further supported liquid EDTA as the preferred final flushing agent for achieving smear-layer-free surfaces prior to obturation [7]. Although gel vehicles offer theoretical advantages of substantivity and sustained contact, the viscosity of gel formulations under sonic activation conditions likely impairs acoustic streaming and cavitation dynamics, thereby limiting the hydrodynamic forces that drive irrigant into dentinal microstructures. This is supported by the intragroup analysis, in which gel groups (1 and 2) showed statistically significant deterioration of smear layer scores from coronal to apical (p = 0.004 and p < 0.001, respectively), whereas solution groups (3 and 4) demonstrated uniform cleaning across all thirds (p > 0.05), a finding that highlights the rheological limitations of gel vehicles during sonic agitation.

The concentration-dependent advantage of 18% EDTA solution over 17% EDTA solution (p = 0.002), especially at the apical third, is supported by the work of Putzer et al., who reported that smear layer and smear plug removal was concentration-dependent, with gels at concentrations ≥18.6% providing better apical cleaning than lower-concentration controls [14]. While the present study did not evaluate dentin erosion, the marginal concentration increment from 17% to 18% may confer a meaningfully higher chelating capacity per unit volume without the structural risk associated with very high concentrations (≥24%). Similarly, the 19% EDTA gel (Group 2) outperformed the 17% gel (Group 1) in overall mean scores (2.28 vs 2.55, p = 0.049), further affirming that concentration modulates efficacy even within gel formulations. This aligns with Kiran et al., who found no significant difference between 17% EDTA solution and 19% EDTA gel under conventional irrigation, suggesting that when activation is controlled, formulation type becomes the predominant determinant of outcome [15].

Sonic activation was employed uniformly across all groups to isolate the effect of irrigant formulation and concentration. The benefit of sonic-activated irrigation over passive needle delivery for chelating agents is well-established: Caron et al. reported that sonic activation with the EndoActivator significantly improved apical third smear layer removal compared to conventional irrigation (p < 0.05) [16]. A study demonstrated that both sonic and ultrasonic activation removed significantly more smear layer than conventional irrigation at the apical third of curved canals [17]. Despite sonic activation, gel groups in the present study still exhibited significantly higher apical smear layer scores than solution groups (Group 1 vs Group 3: p < 0.001; Group 1 vs Group 4: p < 0.001), confirming that activation alone cannot overcome the intrinsic limitations of high-viscosity gel vehicles. Susila and Minu’s systematic review similarly concluded that activated irrigation protocols consistently enhance cleaning efficacy, while acknowledging that the physical properties of the irrigant modulate the ultimate outcome [9]. The consistent finding that the apical third received the highest residual smear layer scores across all groups corroborates the established challenge of apical debridement, attributable to reduced taper, limited irrigant exchange, and the proximity to the apical constriction.

Limitations and clinical implications

As an in vitro investigation, this study cannot fully replicate the dynamic conditions of the clinical environment, including periapical tissue pressure, pulpal blood flow, and salivary contamination. The use of single-rooted teeth with minimal curvature limits generalizability to curved or multi-rooted canals. Dentin erosion and microhardness changes associated with varying EDTA concentrations were not assessed. Clinically, the findings advocate for preferential use of EDTA solution over gel as a final irrigant, particularly in sonically activated protocols, where solution formulations provide more uniform apical debridement. An 18% EDTA solution with sonic activation may offer a clinically meaningful advantage in cases demanding thorough apical smear layer elimination, such as teeth requiring adhesive obturation or regenerative procedures.

## Conclusions

Within the limitations of this in vitro investigation, 18% EDTA solution with sonic activation demonstrated the most effective and consistent smear layer removal across the coronal, middle, and apical thirds of the root canal, outperforming both gel formulations and 17% EDTA solution. Solution formulations exhibited uniform intracanal cleaning, whereas gel formulations showed progressive degradation of efficacy toward the apical third. These findings suggest that when sonic activation is incorporated into the final irrigation protocol, EDTA solution formulations, particularly at slightly higher concentrations, should be the irrigant of choice to achieve comprehensive debridement and optimize the conditions for endodontic obturation.
